# Anthropogenic shift of planktonic food web structure in a coastal lagoon by freshwater flow regulation

**DOI:** 10.1038/srep44441

**Published:** 2017-03-22

**Authors:** Deevesh A. Hemraj, A. Hossain, Qifeng Ye, Jian G. Qin, Sophie C. Leterme

**Affiliations:** 1 School of Biological Sciences, Flinders University, GPO Box 2100, Adelaide 5001, Australia; 2 South Australian Research and Development Institute, PO Box 120, Henley Beach, SA 5022, Australia

## Abstract

Anthropogenic modification of aquatic systems has diverse impacts on food web interactions and ecosystem states. To reverse the adverse effects of modified freshwater flow, adequate management of discharge is required, especially due to higher water requirements and abstractions for human use. Here, we look at the effects of anthropogenically controlled freshwater flow regimes on the planktonic food web of a Ramsar listed coastal lagoon that is under recovery from degradation. Our results show shifts in water quality and plankton community interactions associated to changes in water flow. These shifts in food web interactions represent modifications in habitat complexity and water quality. At high flow, phytoplankton-zooplankton interactions dominate the food web. Conversely, at low flow, bacteria, viruses and nano/picoplankton interactions are more dominant, with a substantial switch of the food web towards heterotrophy. This switch can be associated with excess organic matter loading, decomposition of dead organisms, and synergistic and antagonistic interactions. We suggest that a lower variability in flow amplitude could be beneficial for the long-term sustaining of water quality and food web interactions, while improving the ecosystem health of systems facing similar stresses as the Coorong.

Direct modification of aquatic systems by anthropogenic pollution, or development, has caused diverse impacts on ecosystem functioning and food web interactions^1^,^2^. Some of the most important modifications have involved bottom-up or top-down regulated trophic cascades, significantly influencing trophic interactions in food webs^2^,^3^. Such alterations are often followed by non-equilibrium and degradation (i.e., system recovery is slower than the frequency of disturbance) in ecosystems, therefore, creating instability in ecosystem state^4^,^5^. Unstable systems are often more susceptible to further deterioration in overall ecosystem functional integrity due to quick shifts in environmental conditions and climate change^6^,^7^.

In aquatic systems, the microbial loop plays a highly important role in the regulation of nutrient and carbon cycling, as well as bottom-up control of the food web in an ecosystem^8^,^9^,^10^,^11^. Primary producers are the base of the food web and, therefore closely linked to environmental changes, especially nutrient loading and salinity variations^12^,^13^,^14^. Sudden changes in water quality, such as high nutrient loading, or temperature, are known to shift aquatic food webs by increasing bacteria, viruses and nano/picoplankton activities and biomass^13^, more often creating harmful eutrophic environments than boosting productivity along the food web.

Consequences of food web alteration can be severe for ecosystems, and even more so for those in a non-equilibrium that require longer recovery time. In particular, food web alteration can affect ecosystem functions, thus weakening both food web diversity and economic output^15^. Such situations are typical of many estuaries, where coastal development and pollution have altered ecosystem functioning^16^, and is especially true for the Coorong, South Australia. The Coorong is a shallow saline coastal lagoon of approximately 110 km long which provides a range of commercial and recreational ecosystem services^17^. It is situated at the end of Australia’s largest river basin, the Murray-Darling basin, and is part of a Ramsar listed wetland area (Ramsar Convention on Wetlands, an intergovernmental treaty for the conservation and adequate use of wetlands; www.ramsar.org) called the Coorong, Lower Lakes and Murray Mouth region. The system is only connected to the sea at the mouth of the Murray River and the main source of freshwater is through barrages built to prevent seawater intrusion into the lakes and to regulate freshwater input into the Coorong^18^,^19^.

Freshwater is released into the Coorong in high volumes around colder months and in much lower volumes around warmer months. However, water discharge from the lakes is controlled primarily for the benefit of human use and is dependent on water levels in the lakes. From 2002 to 2009, the system was affected by a severe drought where no water was released and the floral and faunal communities were severely negatively affected^18^,^19^,^20^. Moreover, the ecosystem health of the Coorong was significantly degraded^4^. Since 2010, freshwater release has resumed, but the system remains relatively unstable and under recovery. Moreover, the salinity levels in the southern part of the lagoon remains hypersaline. The availability of freshwater for the Coorong is variable because of natural seasonal fluctuations, but also because of significant water extraction higher up the Murray River for human use. Therefore, the adequate management of water available for release into the system is of high importance for improving the rate of recovery, biodiversity and overall ecosystem health.

In this study, we investigate the effects of freshwater water release on the planktonic food web of the Coorong, to understand the effects of fluctuations in flow regimes on the overall food web interactions. We first look at the communities present in the system along water quality gradients and then examine changes in community interactions to provide an insight into the understanding of the microbial and zooplankton food web, based on flow regime. We hypothesise that the difference in flow regime will cause shifts in trophic interactions. With climate change increasing environmental variability, managers will be challenged to balance freshwater needs of humans and wetlands^21^. This study uses a food web network approach rather than single trophic levels to gain better understanding of ecosystem functioning. This approach was used for the first time on microbial and zooplankton communities in estuaries and coastal lagoons, and provides more applicable information for government agencies to manage water flow and ecosystem health of coastal lagoons such as the Coorong.

## Results

**Figure 1:**
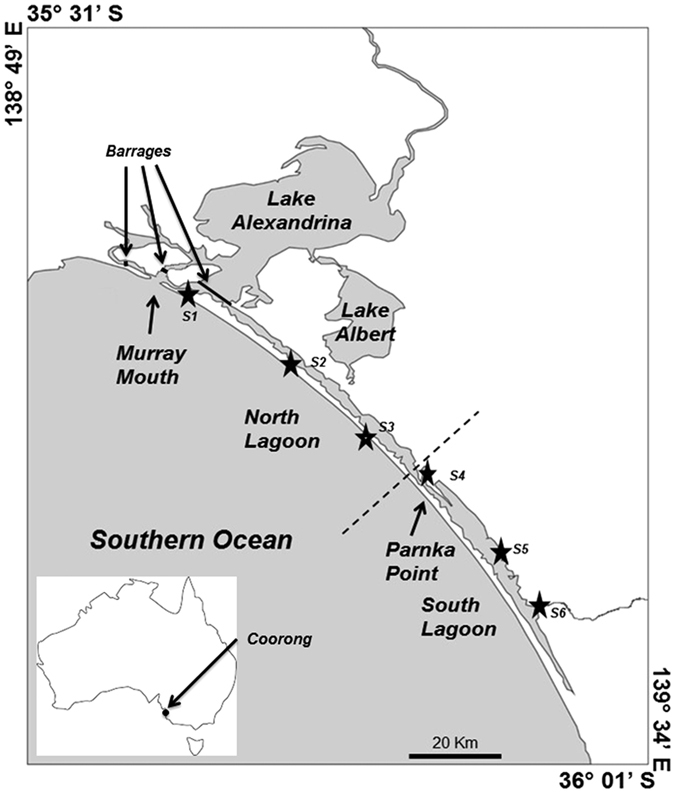
Map of the Coorong and Lower lakes showing lagoons separation and the Murray Mouth. Sampling sites are represented by stars. Barrages regulating the inflow of freshwater are indicated. Basemap was created using ArcMap (version 10.3.3; www.arcgis.com) and Coreldraw X7 (http://www.coreldraw.com/en/product/technical-suite-education-edition/).

**Figure 2:**
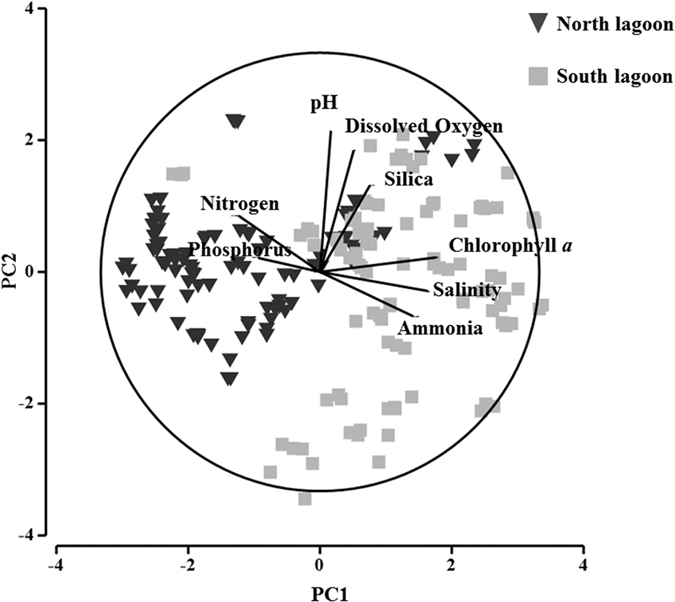
Principal Component Analysis of water quality parameters of the Coorong showing differentiation between the North and South lagoons. The vectors represent the highest contributing parameters (Spearman’s correlation *ρ* > 0.2).

Over the study period, monthly freshwater discharge pulsed from 18,376 ML/Day to 428,472 ML/Day. In the North and South lagoons of the Coorong (Fig. 1), salinity and nutrient levels were the main environmental parameters driving the chemical and biological processes. Flow through the barrages was an important forcing factor driving both salinity and nutrient levels. In particular, flow levels were negatively correlated to salinity (Spearman’s *ρ*: −0.164, *p* < 0.05), especially in the South lagoon (Spearman *rho*: −0.375, *p* < 0.05) and NH_3_ (Spearman’s *ρ*: −0.329, *p* < 0.05), and positively correlated to NO_x_^−^ (Spearman’s *ρ*: 0.309, *p* < 0.05), therefore influencing the nutrient loading into the system. Additionally, the North and South lagoons were significantly different in terms of chemical composition (Fig. 2). The North lagoon varied from low brackish to high marine salinity depending on barrage flow, and distance from the barrages and Murray Mouth. The North lagoon also had significantly higher PO_4_^3−^ and NO_x_^−^ levels than the South lagoon (Kruskal-Wallis one-way: *p *<* *0.05). PO_43_-concentrations remained relatively constant compared to fluctuations in NO_X_- concentrations during the study period, therefore the N:P ratio was more influenced by variations NO_X_- concentrations. Moreover, higher spatial and temporal variability in water quality was observed. On the other hand, the South lagoon was hypersaline throughout the year and it reached up to 98 around summer and autumn, and was characterised by higher turbidity, ammonia levels and total phytoplankton biomass (Kruskal-Wallis one-way: *p* < 0.05).

**Figure 3:**
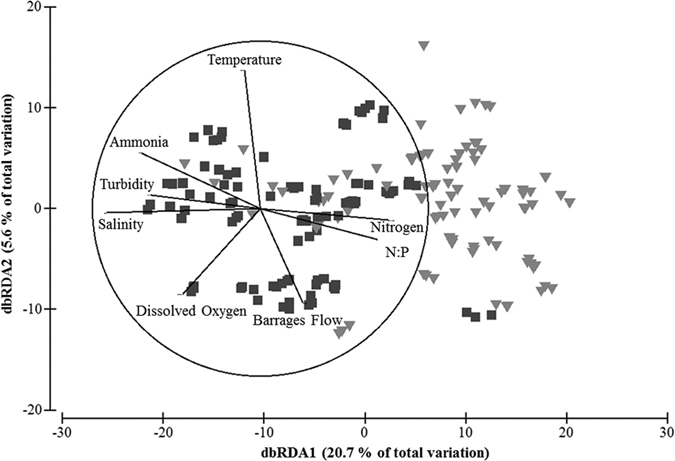
Distance Based Redundancy Analysis of plankton community differentiation in relation water quality changes across the Coorong. The vectors represent the highest correlating water quality parameters (Spearman’s correlation *ρ* > 0.6).

**Figure 4:**
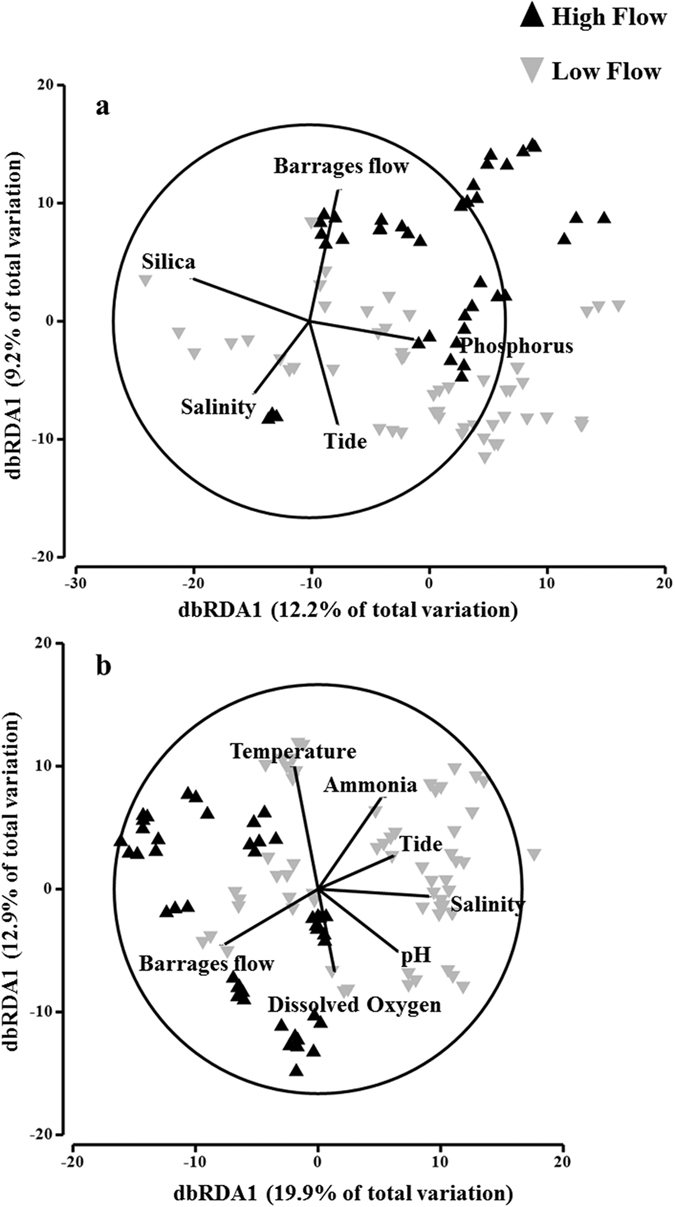
Distance Based Redundancy Analysis of plankton communities in the (**a**) North lagoon and (**b**) South lagoon in relation to water quality parameters and barrage flow as factor. The vectors represent the highest correlating water quality parameters (Spearman’s correlation *ρ* > 0.3).

Different plankton communities were observed between the North and South lagoons of the Coorong (Fig. 3), with water quality being the main driving factors for changes in the communities (Fig. 3). In both lagoons, barrages flow, salinity and temperature were the most important drivers of the fluctuations in overall plankton community (BEST test, *ρ* > 207 and >0.462, for North and South lagoons respectively). In particular, plankton abundances significantly increased with increasing salinity along the system (Spearman’s *ρ* > 0.400, *p* < 0.05), except for zooplankton. In addition, viruses, bacteria, nano/picoplankton, *Prochlorococcus* and *Synechoccoccus* total abundances were positively correlated to NH_3_ levels (Spearman’s *ρ* > 0.280, *p* < 0.05), however, phytoplankton biomass was not. Finally, there was a substantial difference in community structure in relation to barrages flow in both the North and the South lagoons (Fig. 4).

**Figure 5:**
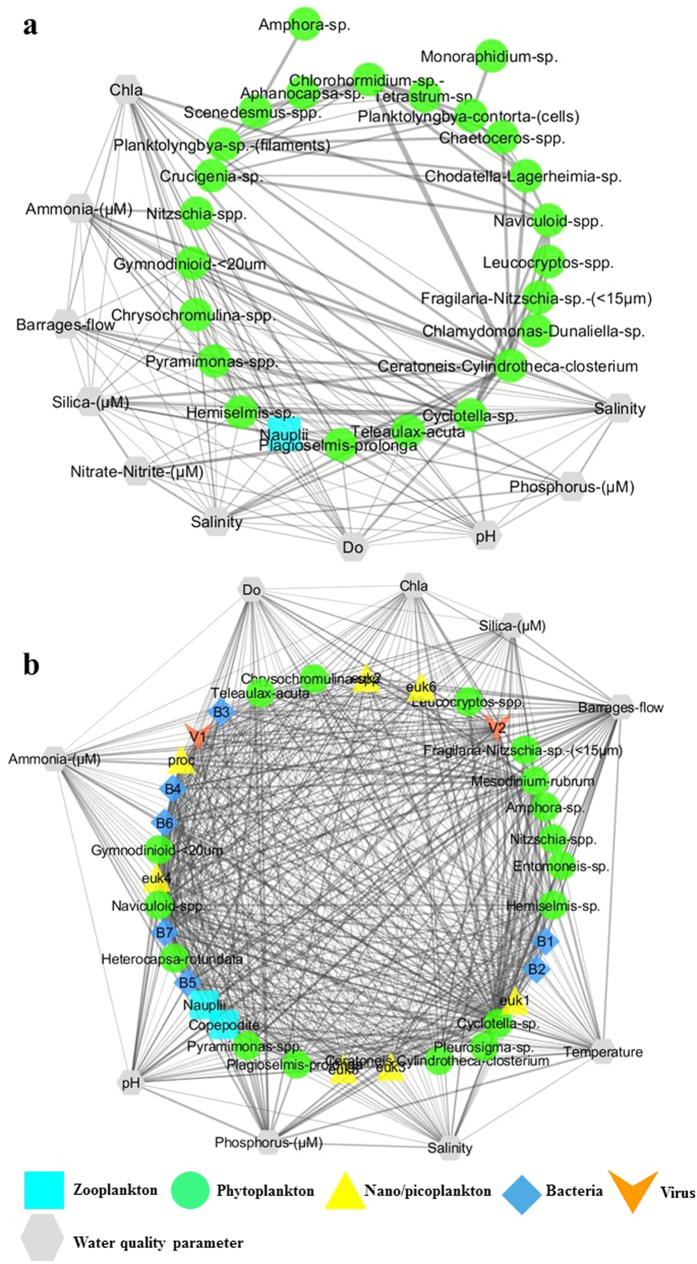
Network of water quality and plankton interactions for the North lagoon of the Coorong under high (**a**) and low (**b**) barrage flow. Edges are undirected and the thickness represent edge-betweenness centrality. A Bray-Curtis similarity threshold of 0.6 was applied to capture the most significant interactions.

**Figure 6:**
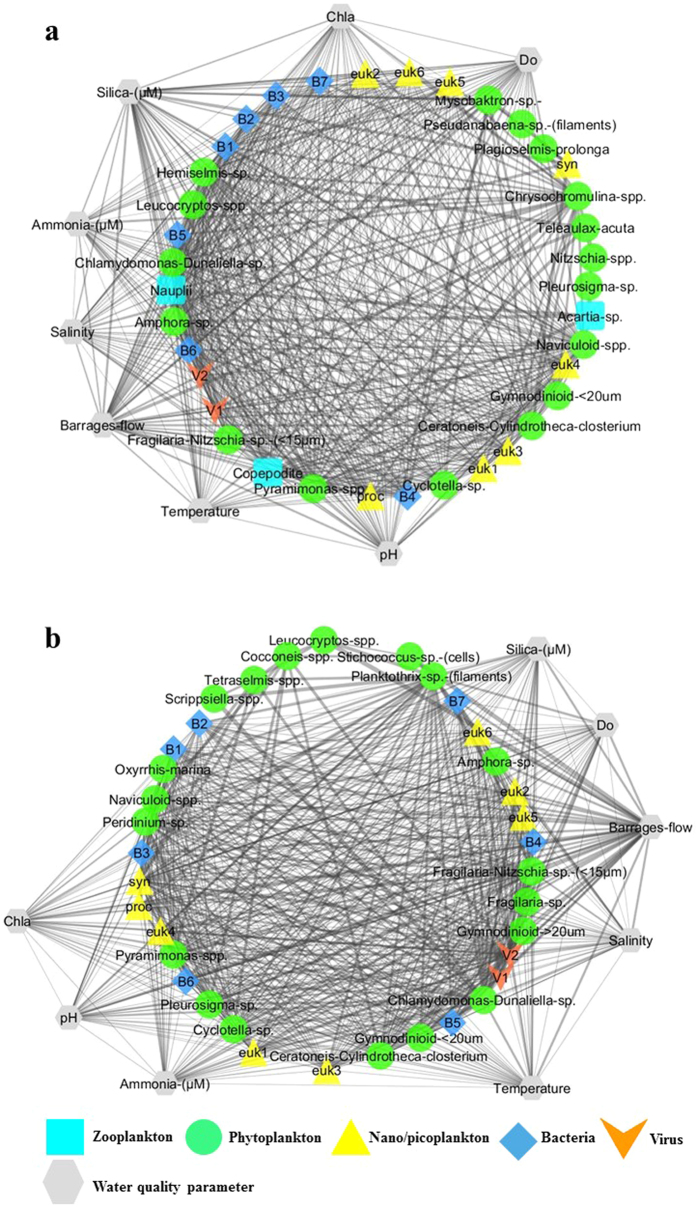
Network of water quality and plankton interactions for the South lagoon of the Coorong under high (**a**) and low (**b**) barrage flow. Edges are undirected and the thickness represent edge-betweenness centrality. A Bray-Curtis similarity threshold of 0.6 was applied to capture the most significant interactions.

Food web network structures were considerably different between the two lagoons, and especially the community interactions between high and low freshwater input regimes (Figs 5 and 6). In the North lagoon, at high freshwater input, the central part of the plankton food web (Bray-Curtis > 0.6) was driven by phytoplankton-zooplankton interactions (Fig. 5a). Moreover, a large number of species were involved (~50% of total number of species observed in the North lagoon throughout the year), all part of either phytoplanktonic or zooplanktonic groups. Closeness centrality values were generally low and spread (0.2–0.6), showing no major control of a particular species on the network. However, copepod nauplii were highly linked to several phytoplankton species (Clustering coefficient^22^: 0.91). Co-occurrence and/or competition for resources between phytoplankton species was also prominent as it’s shown by a large number of linkages between several phytoplankton species in the network. As edges in the network were undirected, the inference of the exact type of interaction is not possible. At low freshwater flow, a significant shift in plankton web interactions was observed (Fig. 5b). Viruses, bacterial and nano/picoplanktonic groups were major interactors in the network (~50% of nodes). Closeness centrality values were also high (0.35–0.87) with viruses, bacteria and nano/picoplankton being integral for the network (Closeness centrality > 0.76).

On the contrary to the North lagoon, at high flow in the South lagoon, the main interactions initially involved viruses, bacteria, and nano/picoplankton communities (Fig. 6a). However, copepod nauplii, copepodite and several phytoplankton species (*Ceratoneis* sp., *Hemiselmis* sp., *Pyramimonas* sp., and *Naviculoid* spp.) still played the central role in the functioning of the network in the South Lagoon (Closeness centrality > 0.81; Network Closeness centrality values: 0.34–0.84). On the other hand, at low flow, similarly to the North lagoon, the food web shifted towards viruses, bacteria and nano/picoplankton dominated interactions (Fig. 6b). The Closeness centrality values of all viruses, bacteria and nano/picoplankton were over 0.86 (Network Closeness centrality values: 0.48–0.89). Moreover, no involvement of zooplanktonic organisms was detected in the network.

## Discussion

Freshwater flow from the barrages is highly important and has a significant impact on the chemical and biological properties of the Coorong. It greatly reduces salinity and NH_3_ levels by encouraging flushing out of the system. Direct flushing mostly occurs for water between the barrages and the Murray Mouth^23^. Most of the flushing for the entire system, however, is a consequence of scouring of the Murray Mouth at higher barrage flow, which then facilitates sea water intrusion and increased water levels, enhancing exchange and mixing between the South and North lagoons^23^. Our results show that the high peak and low trough in freshwater flow through the barrages is highly influential on the changes in water quality. High flow contributes to nitrogen loading, especially in the North lagoon. Nutrient loading into a system alters the balance of nutrient flux and is often linked with blooms of algal species^11^. In the Coorong, however, neither biomass nor abundance of phytoplankton were observed to significantly increase with higher freshwater flow regime and direct nitrogen loading. Excess nitrogen loading through high flow from the barrages increases the nitrogen to phosphorus ratio and intensifies phosphorus limitation, which is likely to prevent excessive algal biomass in periods of higher flow. Moreover, Haese *et al*. showed that there was phosphorus retention in the shallow benthos and that this does not contribute to the phosphorus levels in the water column^24^. This corroborates Aldridge & Brookes’ observations that in the Coorong, phosphorus was low compared to nitrogen in relation to the nutrient requirements of Coorong organisms^25^. Phosphorus limitation during high flow regime, as well as temporal and spatial variations in phosphorus levels, would explain the high importance of fluctuations in nitrogen to phosphorus ratio on driving the plankton community in the North lagoon. Moreover, nitrogen input from barrages does not seem to have an immediate effect on plankton but a rather accumulative and long term effect in case of low flushing out rate.

The South lagoon is composed of different chemical and biological characteristics compared to the North lagoon (Fig. 2). Salinity and NH_3_ levels are the principal driving factors of plankton communities in the South lagoon. Salinity fluctuations in the South lagoon is principally affected by freshwater flow from the barrages that forces exchange and reduces the salinity levels, whereas that of the North lagoon is also influenced by tidal exchange through the Murray Mouth. On the other hand, the high NH_3_ levels are mainly derived from ground water seepage^24^. However, dissimilatory nitrate reduction to ammonium (DNRA) by bacterial populations is also a likely important source. DNRA is an important nitrogen cycling pathway in aquatic systems such as estuaries and salt marshes, and is highly predisposed to a higher carbon to NO_3_^−^ ratio, sulphide level, temperature and salinity^26^,^27^. Such conditions are prevalent in the South lagoon, therefore presenting a highly suitable environment for DNRA. Moreover, bacterial degradation of organic matter is also a likely source^28^. As it’s shown by Fig. 5, prevalence of different environments in the Coorong drives the community change of plankton. Similarly, Leterme *et al*. have shown a split in phytoplankton communities whereby diatoms dominated the South lagoon^19^. Moreover, Balzano *et al*. have shown a distance-driven prokaryote community in the system due to higher variations in water exchange around the Murray Mouth and North lagoon compared to further towards the South lagoon^29^.

Differences in plankton communities imply variations in food web structure. Figures 5 and 6 clearly represent the differences in food web structure between the North and South lagoons. The abrupt change in water quality, related to the variation from a very high flow to a low flow regime, significantly influences the plankton communities (Fig. 4) and seems to drive food webs towards heterotrophy (Figs 5 and 6). In the North lagoon, freshwater input at high flow likely contributes to direct importation of high numbers of freshwater planktonic organisms. Moreover, it contributes to significant organic matter loading^24^ and freshening of the lagoon, therefore modifying the environment^25^. High freshwater pulses have previously been shown to be linked to increased sedimentation, rapid salinity changes, displacement of organisms and shift in community structure^25^,^30^,^31^. Sudden changes to the environment, and to water quality, also create modification in habitat complexity and biotic communities, therefore modifying the degree of species interactions^32^,^33^,^34^. At high freshwater flow, in both North and South lagoons, the food web is primarily dominated by primary and secondary producers. However, once the flow decreases, shift in the species interaction can be observed, whereby the viruses, bacteria and nano/picoplankton become the major part of the web.

Higher involvement of viruses, bacteria and nano/picoplankton in the aquatic food web is often indicative of bottom-up control^10^ and heterotrophy. Moreover, a shift in food web structure can be linked to several processes including response to organic matter loading, bacteria related decomposition, synergistic and antagonistic bacteria and virus interactions, virus-induced prokaryote mortality and predation on prokaryotes by nanoflagellates^22^,^35^,^36^,^37^. For example, Forsström *et al*. have shown that loading of organic matter significantly decreased primary production and caused a shift towards heterotrophic production based food webs, highly influenced by heterotrophic nanoflagellates^38^. Moreover, Sanders *et al*. have also shown a stimulation of heterotrophic interactions by loading of high amounts of dissolved organic matter, but no such effect with moderate loading, whereby primary production was still stimulated^9^. Several other studies have shown changes in specific interactions between bacteria, viruses and nano/picoplankton due to changes in different environmental conditions^22^,^36^,^37^. Although organic matter loading is likely to enhance viruses, bacteria and nano/picoplankton activity, a very high flow is likely to cause substantial change towards heterotrophy, but also light limitation of phytoplankton and benthic algae^9^, therefore also reducing phytoplankton production. Our results also show that, along with changes in salinity and nutrients caused by freshwater discharge, water temperature has an effect on the plankton community interactions (Figs 3 and 4). Increased temperature contributes to higher growth rates of viruses, bacteria and nano/picoplankton and, therefore, contribute to the higher populations observed at low flow. However, although a reduction in freshwater flow into the Coorong occurs around warmer months, the regulation of flow is independent of temperature, but dependent on water levels in the river and lakes. As these tend to be lower during warmer months, we argue that a combination of higher temperature, high organic matter loading and a quick change in water quality due to very low freshwater flow contribute to the change towards a highly heterotrophic system where viruses, bacteria are dominant.

Hydrologic alterations have affected estuaries and aquatic systems globally, especially through reduction of freshwater discharge^39^. The reintroduction of higher freshwater flow is of significant importance for the conservation or restoration of affected ecosystems. However, management of adequate freshwater flow regimes is of equal importance and often neglected in favour of total volume^39^. Sudden transition from low to very high inputs of freshwater, have been linked to changes in net ecosystem metabolism, leading to changes in ecosystem function^40^,^41^. In the Coorong, freshwater release through the barrages is vital for flushing of high salinity and nutrient levels, however, as it’s shown by the networks of species interactions, high and low freshwater inputs are linked to strikingly different food web structures due to the associated changes in environmental conditions (Figs 5 and 6). A reduced difference in amplitude between high and low freshwater flow regimes, that is sustained over an extended period of time, but yet reflecting seasonal fluctuations, is likely to be more beneficial to sustaining the ecosystem’s health. We suggest that such conditions are likely to provide more mixing and flushing out of the system, as well as follow natural seasonal fluctuations in water levels and mixing. Moreover, an extended period of flow is likely to provide a transitional period of change from wet/cold season to dry/hot season, as well as better recovery from the effects of fluctuating water levels and quality. Alternatively, we suggest that a higher monthly minimum water discharge is also likely to increase mixing and flushing out of the system while still following natural seasonal water level fluctuations. This would also reduce the magnitude of stress related to water quality change. Similarly, several authors have argued that increased water discharge could contribute to balance of processes at spatial and temporal scales in systems affected by altered flow regimes, but that each system is likely to behave differently^42^,^43^,^44^,^45^,^46^. However, a higher monthly minimum water release volume would imply an increase in the total volume of water available for the Coorong, which is possibly harder to achieve due to increasing human demands. For the Coorong, although increased water discharge, in terms of a higher monthly minimum volume, is likely to be beneficial, the adequate management of the currently available water may be of similar, if not better, significance. Finally, the implementation of overall network analysis of species interaction in understanding variations in ecosystem function can be a significant tool for managing ecosystems that are anthropogenically modified.

Our results show that pronounced amplitude differences between high and low water discharges may not be beneficial in terms of ecosystem function and the health of the Coorong. Our suggested amendments to water discharge are likely to be highly beneficial for mixing, transitional periods and flushing out of the system, thus improving the recovery rate and ecosystem health of the Coorong. However, further study and monitoring should be encouraged, especially in relation to the hydrodynamic effects and change in overall community interaction that might follow. Finally, with global change influencing environmental variability, managing freshwater needs of humans and aquatic systems is likely to be highly complex. Therefore, adequate use and distribution of freshwater towards the health of aquatic ecosystems under freshwater flow restrictions, or stress, should be further looked into.

## Methods

### Study area

The Coorong is separated from the ocean by sand dunes and the only connection to the sea is at the Murray Mouth (Fig. 1). A natural shallow and narrow channel is present at Parnka Point that separates the system into two lagoons, the North and South lagoons. From November 2013 to October 2014, monthly samples were taken at six sites along the Coorong. Sites 1 to 3 were situated in the North lagoon and Site 4 was situated at Parnka Point, while Sites 5 and 6 were in the South lagoon.

### Water Quality and environmental parameters

Salinity (PSU), pH, temperature (°C) and turbidity (NTU) were measured using an AquaRead multi-parameter probe (Aquaprobe AP-800). Dissolved oxygen (DO) was measured using a Thermo Scientific portable meter (Orion Star A323 RDO/DO). Triplicate water samples (10 mL) were collected at each site for the determination of dissolved inorganic nutrients concentrations including silica (SiO_2_), ammonia (NH_3_), orthophosphate (PO_4_^3−^) and nitrate/nitrite (NO_x_^−^) and analysed following Leterme *et al*.^19^.

Flow data for the barrages and Salt Creek were sourced from the WaterConnect website (www.waterconnect.sa.gov.au) and from the Department of Environment, Water and Natural Resources (DEWNR).

### Viruses, Bacteria and Nano/picoplankton

Triplicate samples of 1 mL were taken at each site for the analysis viruses, bacteria and nano/picoplankton populations. Viruses and Bacteria samples were preserved with 2% glutaraldehyde and nano/picoplankton samples were preserved with 12.5% paraformaldehyde. Samples were frozen in liquid nitrogen and stored at −80 °C until analysis. The analysis of samples were carried out using a BD FACScanto 2 flow cytometer following the protocol by Marie *et al*.^47^.

### Phytoplankton and Zooplankton

Phytoplankton samples were collected using a Niskin bottle and preserved in 5% Lugol’s solution. Identification and enumeration of samples were carried out by microscopy at Microalgal Services in Ormond, Victoria as described by Leterme *et al*.^19^. Zooplankton samples were collected using a modified Schindler-Patalas trap. Samples were preserved in 2 to 5% formaldehyde. Identification to the lowest taxonomic level and enumeration were carried out by inverted microscopy.

### Statistical analysis

Data analysis was carried out using SPSS and Primer + Permanova. All data were tested for normality prior to analysis. Correlations coefficients between biotic and abiotic data were calculated using Spearman’s correlation. Significance in difference in data were tested using Kruskal-Wallis one-way ANOVA. A Principal Component Analysis (PCA) was used to explore the water quality parameters contributing to the differentiation between the North and South lagoons. Biotic data were log (X + 1) transformed and a Distance Based Redundancy Analysis (dbRDA) was used to explore the effects of environmental parameters on differentiating the planktonic communities along the system. A Spearman’s correlation threshold of 0.6, was applied to plot the most highly correlating variables. Moreover, two separate dbRDAs were used to look at the community of the North and South lagoons, separately, in relation to barrage flow. Finally, a BEST (BIOENV) test was used to identify the most important environmental parameters influencing the plankton communities in the North and South lagoons separately.

Analysis of species interaction in the planktonic food web was carried out by network analysis using the free software package Cytoscape (http://www.cytoscape.org/)^48^,^49^. Networks were created to identify community interactions under a high or a low freshwater input into the Coorong, using Bray-Curtis similarity. A threshold of 100,000 GL was used to differentiate between high and low freshwater inputs. Also, a Bray-Curtis similarity threshold of 0.6 was used for all networks to capture the more significant community interactions. The threshold allowed for the exclusion of outliers. Analysis of network was done using the Network Analyser application^50^. Closeness centrality and edge-betweenness were implemented in the layout of network (Closeness centrality and edge-betweenness are network specific). Closeness centrality denotes the probability of a node to be functionally relevant for several other nodes, therefore can be central for the regulation of other nodes. Closeness centrality values range from 0 to 1, where 0 is low and 1 is high. Edge-betweenness denotes the importance of the interaction between two nodes for the network functional organisation. The higher the edge-betweenness, the more important the interaction is for organising other processes in the network^51^,^52^.

## Additional Information

**How to cite this article:** Hemraj, D. A. *et al*. Anthropogenic shift of planktonic food web structure in a coastal lagoon by freshwater flow regulation. *Sci. Rep.* **7**, 44441; doi: 10.1038/srep44441 (2017).

**Publisher's note:** Springer Nature remains neutral with regard to jurisdictional claims in published maps and institutional affiliations.
